# The Early Modern Silesian Gallows (15th–19th Century) as an Example of Stray Animals Utilization before the Rise of Institutional Veterinary Care

**DOI:** 10.3390/ani11051210

**Published:** 2021-04-22

**Authors:** Aleksander Chrószcz, Dominik Poradowski, Paweł Duma, Maciej Janeczek, Przemysław Spychalski

**Affiliations:** 1 Division of Animal Anatomy, Department of Biostructure and Animal Physiology, Wrocław University of Environmental and Life Sciences, ul. Kożuchowska 1, 51-631 Wrocław, Poland; aleksander.chroszcz@upwr.edu.pl (A.C.); maciej.janeczek@upwr.edu.pl (M.J.); przemyslaw-spychalski@wp.pl (P.S.); 2 Institute of Archaeology, Faculty of Historical and Pedagogical Sciences, University of Wrocław, ul. Szewska 48, 50-139 Wrocław, Poland; pawel.duma@uwr.edu.pl

**Keywords:** gallows, animal, knackers’ yards, waste pits, utilization, dogcatcher

## Abstract

**Simple Summary:**

The history of veterinary medicine starts from ancient times. The lack of professional veterinarian education and institutional veterinary services from the Middle Ages to modernity resulted in, partially, a basic realization of these duties by blacksmiths, herders, butcher guilds, municipal doctors, and executioners. Therefore, the archaeological excavations carried out at gallows in three towns of Lower Silesia, together with the archaeozoological analysis of unearthed animal skeletal remains, brought valuable information on human–animal–environment relationships in the past. This includes the role of an executioner or a knacker (in animal population control), the utilization of stray animals, and the use of animal products accessible in a knacker’s yard. This work highlights the history of early modern towns that attempted to solve the problems surrounding the lack of veterinary care, before the rise of modern professional veterinary education, and the introduction of the veterinarian profession in modern human society. The results show that the majority of investigated animal skeletal remains came from adult and healthy animals, and after animal death, some parts of animal bodies were used (e.g., leather, fat, bones) by knackers as sources of additional profits, but the main sanitary problems could not be permanently eliminated.

**Abstract:**

In the past, executioners played an important role in the legal system. Besides sentence executions, they also worked as dogcatchers (i.e., eliminating stray animals or cadavers of dead animals from towns), and were responsible for sanitary conditions within their towns and closest neighborhoods. Archaeological explorations of gallows in the towns of Lower Silesia (Poland) provide evidence of such activities, including animal skeletal remains. Archaeozoological analysis of these materials from the towns Kamienna Góra (Landeshut), Złotoryja (Goldberg), and Jelenia Góra (Hirschberg) are the subjects of this study. Our work also stresses the nature of the executioner’s profession in animal health control and town hygiene maintenance before the development of modern veterinary services. The results show significant differences in the frequency of species and distribution of anatomical elements in accessible assemblages compared with animal skeletal remains unearthed in typical waste pits or classical inhumation, allowing the assumption that the animals were anatomically adults, and their health statuses were generally good. The dominant species, equids and dogs, were represented by skeletal remains, with the predominance of less valuable body parts (distal parts of appendices, caudal parts of the vertebral column). The fragmentation of accessible bone assemblages narrows the ability of larger conclusions (i.e., minimum number of individual estimations). The work enlightens the complex role of executioners pertaining to the hygiene of early modern town communities, a role later replaced by professional veterinarians with all of the consequences of the transition process.

## 1. Introduction

Scant animal skeletal remains dating back to the Mesolithic period are rarely presented in literature and, therefore, this historical period is not often the focus of interest in zooarchaeology [1,2]. The earliest (and broader) descriptions of human–animal–environment relationships in the past were commonly built on zooarchaeological analyses of animal wastes from archaeological sites, dating back to the Neolithic period [3,4,5,6]. Animal waste pits containing skeletal remains became a constant feature identified in archaeological explorations. The majority of them date back to the Neolithic, Bronze, Iron Age, or Medieval times; these periods used to be the most frequent purposes of archaeozoological investigations [3,4,7,8]. The need for scientific elaboration of later post-medieval animal bone deposits is an important source of information on sanitary and economic regimes; the use of animals; attitudes to animals; and the symbolic role of animals as food, was emphasized in Britain by Thomas [9]. Recent historical periods were rare, and did not elaborate on human–animal–environment relationships based on archaeological and archaeozoological sources from Poland, in contrast to Western Europe [10,11,12,13,14,15]. The need for such studies, and their valuable contributions, to our knowledge, about the mentioned relationships in the past, was highlighted in literature [16].

The rise in domesticated animals led to an increase in the development of animal bone waste recycling. This is evident because of the changes that can be seen in the bone structures of the animals as they evolved from wild to domestic animals. In addition, the markings of the bone fragments provide evidence of diseases caused by inbreeding and overloading working animals that were used to pull carts and farming implements. The most frequently described specimens were identified as animal skeletal remains, the result of an increased consumption of animal meat (widespread from the Neolithic to Middle Ages), or rarer specimens, but more spectacular was the discovery of animal bone tool findings [3,8,17].

The development of ancient medical and veterinary sciences resulted in the increased application of more formal regulations for the slaughter of animals and the trading of animal products; this became especially efficient during the Roman Empire. The fall of the Western Roman Empire and the beginning of the Middle Ages eradicated the use of any universal regulations regarding the use of animals and the connected trades. Local rulers sent out orders complying with local requirements, but instead of using qualified veterinarians, animal care was provided by herders, shepherds, breeders, blacksmiths, or executioners [17,18,19]. The animal care that was provided was much more primitive than it is today. It was possible to understand the treatments, breeding methods, and use of animals from some fragmentarily-preserved Ancient or Moorish text translations, which were more popular during the Renaissance Period, but were mainly based on evidence of human practices (e.g., blacksmithing, including skillful horseshoe construction), traditional skills passed from generation to generation using the spoken word (master–pupil relationship), as well as superstition practices (e.g., using feces for medicinal purposes). Despite these human interventions, it was not possible to provide the level of medical or surgical treatments, infectious disease controls, animal use controls, or welfare that is available today. There was an attempt by the Holy Roman Emperor Frederic II Hohenstaufen to create universal regulations of animal slaughtering that could be controlled by law, in a ruling issued on 8 June 1231. However, it was practically impossible to enforce this ruling outside the territories directly dependent on the sovereign [18]. The Municipal Magdeburg law (*Ius municipale magdeburgense*) was introduced the first time in 1188 for the city of Magdeburg by archbishop Wichmann of Seeburg, and later widespread in Medieval Middle Europe. It was known as the German Municipal Law in Silesia and Moravia and it regulated the majority of city life. Złotoryja was the first town in Silesia located in 1211. The located town consisted of the market place with the town hall, regular street orientation, and parceling of ground within the city walls. The town council, town mayor, and masters of guilds were responsible for town trade, craft, and town court. The evolution of urbanization resulted in the gradual changes of town funder/owner—town patriciate member relationships. Right swords (*Ius gladii*), a legal power of attorney to pronounce the death penalty (primarily reserved for feudal masters) were ceded to town councils. This process changed the role of qualified executioners and their positions in medieval and renaissance social hierarchy [20,21,22]. The profession became very important in medieval and early modern communities, as it alleviated the problem of stray and useless animals. It therefore seems interesting to analyze the role of early modern executioners and gallows based on archaeozoological findings. Although the standard locations of gallows in European landscape are well known [23,24,25,26,27], it is rare to find the structural relics of gallows persevered in Poland [28].

Finally, the increase in the population of modern Europe, and the consequential increase in the concentration of domestic animals, and animal plagues, together with livestock trade and migration, called for a more inclusive and comprehensive veterinary service to be available. The increasing quality and value of the domesticated animals and the intensification of animal production, nutrition, and management resulted in the development of modern veterinary education in the 18th and 19th centuries. Students attending veterinary medical schools became the first people to deal with animal plagues and zoonoses [17,29] (diseases that could be transmitted to humans from animals). The development of veterinary intervention, animal product inspection, and health management in animal production brought about important changes in the legal system. Moreover, veterinary doctors started to play a crucial role in public health management [17,18,29]. Early modern municipal executioners controlling the animal population ceased to operate, but signs of their activities can be identified and described by archaeologists analyzing the rare discoveries when gallows were unearthed.

The primary aim of this study was to fill the gap in the literature concerning the lack of wider archaeozoological elaborations on animal bone remains dating back to Early Modern Times, and the unique findings of a complete animal catcher (knacker) and animal waste pit located within the gallows unearthed in southern Poland encouraged us to undertake this study.

Secondarily, this work became a rare opportunity to describe the role of executioners and knackers’ yards in early modern municipal centers of Lower Silesia, before the development of professional veterinary animal healthcare, supervision, and sanitary control.

### 1.1. Archaeological Sites

The archaeological exploration focused on three archaeological sites located in Lower Silesia (Poland) and recognized as former gallows and knacker’s yards. The first was located in Jelenia Góra (Ger. Hirschberg), and is known as the new gallows. The next two sites were in Kamienna Góra (Ger. Landeshut) and in Złotoryja (Ger. Goldberg). Each site represents the typical location and construction anticipated. They were located outside the city walls, in a clearly visible elevated area. The central part was a cylindrical construction with pillars and a crossbeam at its crown, surrounded by the knacker yards.

The Kamienna Góra gallows and knacker’s yard functioned between the 15th and 18th centuries. Both in the vicinity, as well as in the interior of the gallows, numerous animal bones, with a small share of human bones, were found. All skeletal fragments were disarticulated and damaged. Animal bones were found in a small feature (1 × 1.2 m, 0.55 depth) discovered below the former place of execution and also in disturbed top layers. The most interesting discoveries were found in an oval-shaped animal catcher’s waste pit (no. 1/13) located close to the gallows (measured 3 × 2 m, 1 m depth). The pit contained numerous animal bones, a few human ones, and several artefacts. Based on recovered pottery fragments and clay tobacco pipe analysis, it was possible to date the structure to the first half of the 19th century. Pipe fragments originating from Zborowskie were found, where the manufacturing of pipes existed between 1752 and ca. 1820. According to the analysis of textual sources from Złotoryja, the former knacker’s yard area was located at some distance from the execution site. Archaeological excavations, however, proved that animal remains were deposited inside the gallows. The long-term deposition of waste, bones, and naturally deposited material inside the gallows between the 15th and the beginning of the 18th century, led to the creation of layers, with a total thickness of approximately 80–90 cm. The excavations exposed animal remains mixed with human bones and artefacts. All the bones were disarticulated. In other trenches located outside the gallows’ foundation, only a few bones were found. In Jelenia Góra, all of the animal bones were found in a layer directly beneath the demolition layer and they were left between 1778 (when the gallows were erected) and 1834 (when the gallows were demolished). According to textual sources, we know that within the execution site borders, a knacker’s yard also functioned. The site was used for a relatively short period of time compared to other sites. During the excavation, hundreds of animal bones were found inside the gallows, but there were no human bones among them [20,28,30]. Złoty Stok (Ger. Reichenstein) was mentioned only as a digression and example of the cat skeleton deposition placed within the former gallows [28].

### 1.2. History of Sites and Archaeological Context of Animal Remains

The history of Silesia was especially stormy and this had an impact on the role that Kamienna Góra, Złotoryja, and Jelenia Góra, three medium sized towns of the Silesian province, played in this area of Poland at the time. There was a period of alternating supremacy between the Polish Piast and Czech Přemyslid Dynasty, followed by the Silesian Duchies, subsequently swearing homage to, or being incorporated into the Kingdom of Bohemia, and together it became part of Luxembourg, Jagiellonian, and Habsburg monarchies. These times are linked with the oldest part of archeological findings dating back to the 15th century. During the Thirty Years’ War (1618–1648), Silesia was constantly under attack from the Catholics and Protestants. Three Silesian gallows were found and evidence suggests that their purpose was not solely to be used as the places for the administration of justice, but also as knacker’s yards. The Silesian Wars (1740–1763) established the province of Silesia within the Kingdom of Prussia and, subsequently, from 1871 it became part of the United German Empire of Hohenzollerns [31,32,33].

This, together with the orders from the new rulers, meant that gallows gradually vanished from the Silesian landscape, and were replaced by traditional knacker’s yards. The development of the modern veterinary service and an adequate legal system, which included animal slaughter and food control, was introduced in Prussia in 1844. Wroclaw (Ger. Breslau) authorities controlled all of the municipal slaughterhouses in this area from 1858. Finally, the introduction of unified sanitary and veterinary law in the German Empire in 1878 excluded the former executioner from his duties and his responsibility for the ways in which animals could be used [18].

**Figure 1:**
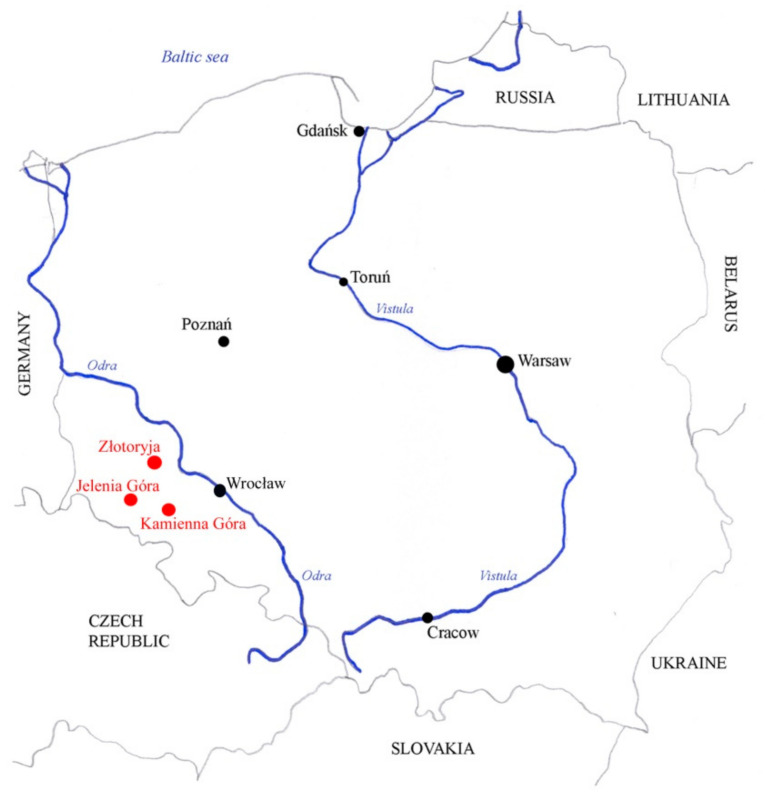
Locations of archaeological sites (red points). Map presents main cities (black points) and main rivers (blue lines) by A. Chrószcz.

Former execution sites located in Lower Silesia (Poland) were investigated for a decade [28]. Skeletal material were unearthed during archaeological excavations carried out in 2012–2014 by the Institute of Archaeology, University of Wrocław, in three Silesian towns: Kamienna Góra, Złotoryja, and Jelenia Góra (Figure 1).

## 2. Material and Methods

The bone remains were divided into groups, of which visual-comparative identification revealed the total number of fragments (TNF) and number of identified specimens (NISP) in animal skeletal remain assemblage (Table 1) as follows:

1. First group: skeletal remains from Kamienna Góra gallows functioning in the 15th–18th centuries, including a complete animal-catcher waste pit (no. 1/13), finally closed in the first half of the 19th century.
2. Second group: skeletal remains from Złotoryja dated between the 15th and 18th centuries.
3. Third group: skeletal remains from Jelenia Góra dated between the 18th and 19th centuries.

During the visual-comparable analysis, both epiphyses and diaphyses of the long bones were used and the measurable identified specimens were collected for osteometry. The animal age was estimated on the basis of the epiphyseal cartilages existence and the lack of fusion between the long bone diaphysis, epiphyses, and apophyses [8,22,34,35,36,37]. The minimum number of individuals (MNI) was estimated on the minimal number of examples (MNE). Due to the poor preservation status, the achieved results have only approximate meaning [38].

The pathological changes visible in archaeozoological assemblage were identified. A wider description and explanation of potential causes of pathogenesis, symptoms, and prognoses will be the aim of separate work.

The last stage of the archaeozoological analysis was aimed at describing the human–animal relationship and animal waste utilization methods in the gallows of early modern Lower Silesian towns [28,39,40]. The development of early modern veterinary service and duty was linked and confronted with an earlier way of the animal problem solution, which became insufficient at the beginning of modernity.

The results are presented in the tables and figures. Photographic documentation of the investigated material was performed.

## 3. Results

**Figure 2:**
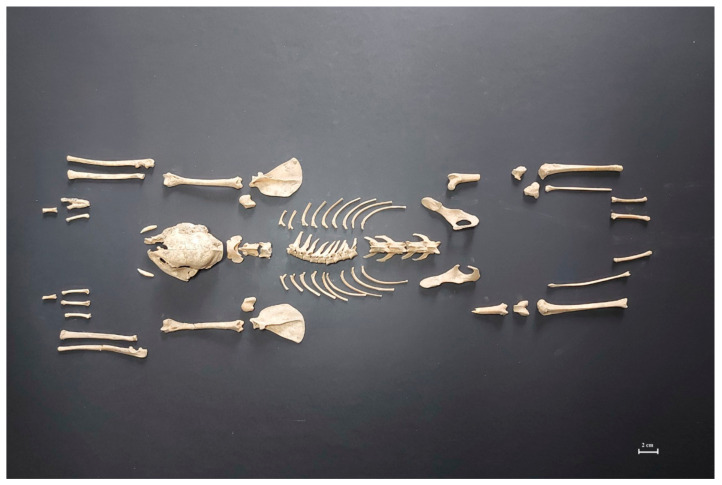
Incomplete domestic cat skeleton unearthed at Złoty Stok gallows site as the example of only discovery that was accessible for archaeozoological analysis.

The visual-comparative analysis showed that the total number of fragments from Kamienna Góra and Złotoryja were similar, but NISP in Kamienna Góra was almost two times lower than in Złotoryja (Table 1). The third archaeological site (Jelenia Góra) showed the highest percentage of NISP as Złotoryja and Kamienna Góra. The total number of bone fragments in Jelenia Góra was the smallest, while the NISP at this site was the highest. The animal bone assemblages unearthed in Jelenia Góra gallows were scarce. They came from the old gallows that were permanently closed in 1778 and replaced with a new one, later transformed into a classic knacker’s yard. The latter process was clearly visible in the skeletal waste assemblage from Kamienna Góra gallows. The area was rich in both human and animal bones, with a high percentage of burned artifacts. The percentages of NISP and burned bones in Kamienna Góra and Jelenia Góra were similar (Table 1). Therefore, we can state that small Jelenia Góra bone assemblage species composition corresponded with a much larger one excavated in Kamienna Góra—distinctive for such findings dating back to a similar time period and geographic location. Since the bones were burnt, it is likely that the bones would have been buried immediately after burning and not piled in place, and only deposited during infrequent upkeep of the site. The written sources proved that periodically, before the important officials visits, the knacker’s yards and gallows neighborhood were cleaned, and all skeletal remains utilized (burned and deposed in accessible pits [28,30]. The gallows existence in Złoty Stok was proved during an archaeological exploration. The minimal number of animal bone remains (only one incomplete skeleton of a domestic cat was found), was explained by later use of the investigated area as a farming field (Figure 2) [28].

At all investigated sites, the majority of the identified skeletal material came from domestic animals. Table 2 presents the frequency of species at the analyzed sites. All three animal bone assemblages (Kamienna Góra, Złotoryja, and Jelenia Góra) showed the highest percentage of equine and canine skeletal remains. The skeletal remains of other domestic mammals and humans equals 5–8% of NISP. A detailed analysis of human bones will be presented in a separate work, as it is not the aim of this study.

Due to the poor preservation status of accessible material, the minimum number of individuals (MNI) estimation was presented as approximate values in Table 3. The strong fragmentation of axial skeleton and long bones, numerous independent dental fragments, or the high quantity of phalanges and sesamoid bones caused widespread ranges of potential MNI values. Therefore, the MNI quantitative analysis was used only for long bones, calcaneus, and talus. The mentioned values were estimated for two of the most numerous specimens (horses and dogs). It seems that the mean values of the latter parameter will be useful for readers in imagination of carcass potential quantity.

**Figure 3:**
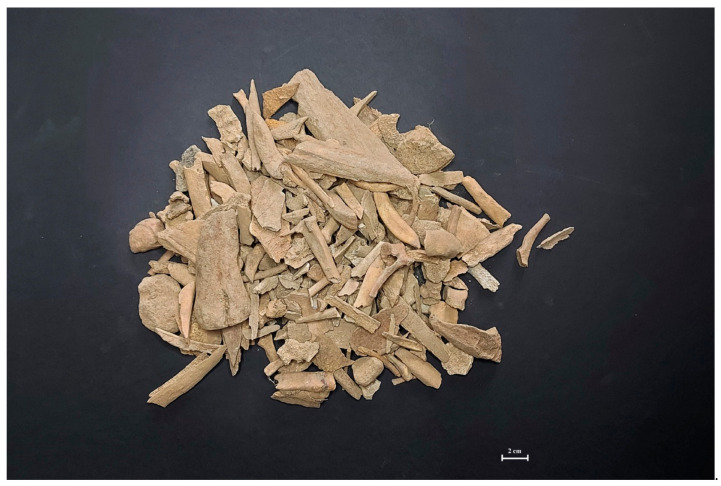
Typical of animal bone assemblage from Kamienna Góra site.

The morphometric analysis of investigated assemblages was possible only in the bone remains form Złotoryja and Kamienna Góra. Strong fragmentation of accessible material (Figure 3) excluded this stage of archaeozoological analysis in many cases. The achieved results were shown in Table 4, Table 5 and Table 6.

The distribution of anatomical elements is a useful tool in archaeozoological analyses. It is based on the animal bone remains divided into groups corresponding with subsequent body parts: head, trunk, proximal and distal appendices, and phalanges. Normal distribution of anatomical elements was defined experimentally and presented in body part percentage of the whole skeletal system. It can be observed in causes of burials. The differences, the deficiency, or excess of exact group (body part) can be observed when the bones forming the body part are less or more numerous [37].

The distribution of anatomical elements of horse and dog skeletal remains are presented in Table 7.

**Figure 4:**
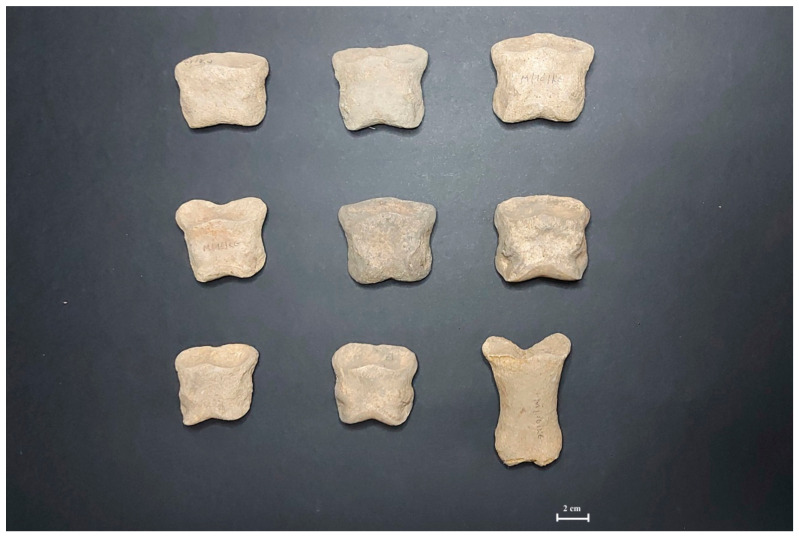
Horse phalanges discovered at Kamienna Góra site.

The distribution of anatomical parts of horse skeletal remains from the Kamienna Góra gallows shows a significant deficiency of trunk and proximal appendices skeletal elements, together with an excess of distal appendices skeleton and phalanges (Figure 4).

Contrary to that, the archaeozoological assemblage from Złotoryja comprised of an excess of trunk skeleton elements and a deficiency of head skeleton elements, and from Jelenia Góra, a deficiency of head skeleton parts and an excess of phalanges. The distribution of anatomical elements of canine skeletal remains was similar. At all three sites, we observed an excess of appendices and a deficiency of head and phalanx skeletal elements. The investigated bone assemblages differed in the number of trunk skeletal elements. It was similar to the normal distribution of anatomical elements in Kamienna Góra, while a small deficiency was observed in Złotoryja and an excess of these skeletal parts in Jelenia Góra.

## 4. Discussion

Detailed archaeological explorations of early modern gallows unearthed in Poland were rare. Therefore, the archaeozoological studies of gallows and knackers’ yards from this part of Europe are especially important [24,25,26,27,28,41,42].

### 4.1. Osteological Analysis

Frequency of species at all three sites (Kamienna Góra, Złotoryja, and Jelenia Góra) did not resemble the most frequently identified consumptive waste identified in settlement areas from Modern World in Poland, predominantly, remains of cattle and small ruminants were found [43,44,45,46,47,48]

As for wild animal remains, the most interesting is the presence of snails and other wild animals (i.e., fox and birds) in Złotoryja, but not in Kamienna Góra or Jelenia Góra (marginal intake of wild-living animals). The presence of these species can be explained by some artefacts, which occurred accidentally and were within knackers’ yards. The snail species represented in the analyzed assemblages were probably one of the local species naturally occupying these areas. It is also possible that they occurred in high numbers due to the favorable “micro-ecosystem”—a high supply of decomposing organic matter (animal carcasses) or vegetation growing nearby. Moreover, the presence of skeletal remains coming from wild boars, hares, or other rodents can be explained by the normal habitat of mentioned species, ubiquitous, and able to live both in forests and fields, and close to human neighborhoods. The available literature shows that wild animals and even domestic dogs were frequently found at typical gallows due to their alimentary habits and interests [23,24,25,26,27,28]. The lack of such findings in Kamienna Góra and Jelenia Góra could be due to a greater share of burned bones in this assemblage (Figure 3). These facts indicate possible large-scale skeletal waste utilization and potential re-deposition, also limiting our ability to identify bone fragments. The historical archives bring some information about periodical gallows, knackers’ yards, and direct neighborhood cleaning by executioner’s peon, ordered by municipal authorities, prior important events, or rulers’ visits [49]. The proof of similar, intentional human activities aimed at animal bone wastes were described during the archaeozoological analyses of animal skeletal remains unearthed in other sites identified as gallows and knackers’ yards [23,42].

It should also be noted that the presence of human bones in the analyzed assemblages can be explained by a custom of abandoning hanged cadavers until their total disintegration, as a sign of juridical power and convict disgrace, and as a warning to other people [21,23,25,26,27,28,39,40]. At the same time, historical information on the executioner’s practices concerning human burial conventions in the neighborhood of the gallows was not fully evidenced in various places of Lower Silesia [39]. The latter hypothesis can be supported by the findings of other authors. Auler [42], in the archaeological results summary from gallows in Switzerland, showed hard evidence that human and animal skeletal remains existed within the places of execution, from over 600 individuals in Emmenbrücke to less numerous finding in Willisau-Steinmatt. Moreover, the majority of animal bone waste from Emmenbrücke were recognized as coming from horses and dogs [23]. Both authors stressed that the executioners of early modernity were also responsible for the collection and utilization of stray animals and cadavers.

Full exploration of the animal catcher’s waste pit (object no. 1/13) seems unique, not only because it is the first discovery in Poland, but also that it allowed us to perform a detailed analysis of this extraordinary bone assemblage (Figure 3). Frequency of species was similar to that in Kamienna Góra and Jelenia Góra, with the highest percentage of equine and canine bone remains and minimal intake of wild-living species. It seems that a full exploration of such archaeological features could be representative of the entire process for how the knackers’ yards and gallows’ neighborhoods were used in the context of animal bone remains.

Due to strong fragmentation of the investigated material, its morphometric analysis was considerably limited. The animal height at the withers fits the range of mean values for all species. The scarcity of measurable bones limits further interpretation and conclusions to be drawn from the skeletal material from Kamienna Góra and Złotoryja (Table 4, Table 5 and Table 6).

The height at withers was computed on the basis of accessible bone remains for horses and dogs [50,51]. The mean values of the mentioned parameter resulted in measurements of 133.1 cm and 135 cm for horses from Kamienna Góra and Złotoryja, and for dogs 33.3 cm in Kamienna Góra and 46.2 cm in Złotoryja. The horse’s height at the withers is similar in both cases and can be described as smaller than modern horses (ca. 20–30 cm) [52]. During the time-consuming studies of horse skeletal remains from medieval Wrocław (Silesia), Chrzanowska [53] defined four groups of horses, based on height, at the withers. The achieved results allow for classification of the horses from Kamienna Góra and Złotoryja to small and medium size. The height at the withers computed on the basis of horse bone remains from Zürich-Albisrieden were similar or slightly greater [24,25]. This medium-to-small body size could be explained, not only by the animal breed, but also by the environmental pressure, which caused limited expression of genotype (phenotype). The dog remains analysis showed a larger range of values of investigated parameters (from 27.6–47.2 cm), comparable to modern small- and medium-size breeds. The maximum dog’s height at the withers was similar to the mean value computed for this parameter in late medieval Wrocław [45]. Their body sizes resemble the animals from the same city, but dated back to the 13th century [54].

The distribution of anatomical elements of animal skeletal remains further confirmed the above-stated thesis. The distribution of anatomical elements of bone remains together with the frequency of species from the analyzed archaeozoological sites (Table 2) differed greatly from normal distribution of anatomical elements (usually observed in causes of burials) and typical species composition for consumptive animal bone waste (predominance of cattle, swine and small ruminants) [8,24,55]. As two of the most numerous genera were horses and dogs, we narrowed down our investigation to these animals (Table 4). Similarly, the predominance of horse and dog skeletal remains was proved in Emmenbrücke [23]. Other species of domestic mammals and human remains did not exceed 5–8% of NISP, indicating that the executioner activities mainly involved utilization of equine and canine cadavers.

**Figure 5:**
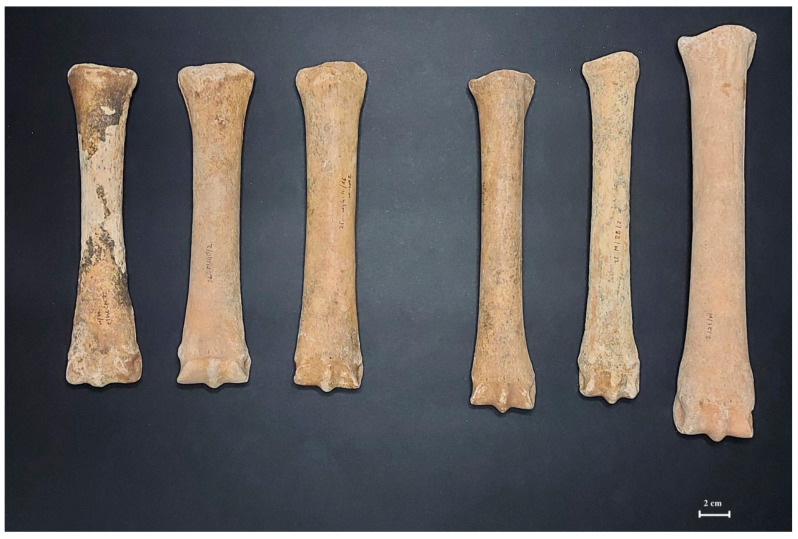
Horse metapodial discovered at Złotoryja site.

The excess of horse’s trunk skeleton elements is typical for classical consumptive materials, highlighting an important difference compared with animal bone assemblages from the knackers’ yard (Table 7). The typical consumptive skeletal remains assemblages are characterized by clear predominance of domestic species, such as: cattle, pig, sheep/goat group, etc., as the most frequent source of animal meat for humans from Neolith to Early Modern Times [5,43,44,45]. Such archaeozoological analyses proved that the bone remains of horses and dogs usually do not exceed ca. 3% of NISP for horses and ca. 5% of NISP for dogs. Simultaneously, the horse’s skeletal remains classified as inhumations show the lack of other taxa, what can be interpreted as a result of intentional human activity [55]. Moreover, in this study, we observed a clear predominance of caudal vertebra remains versus others of the vertebral column. This can be explained by the fact that, together with the great number of phalanges and distal appendices, the skeletal remains of horses were treated as waste and unfit from a culinary point of view (Figure 4 and Figure 5).

More attractive equine body parts were not deposited there and the presence of phalanges can indicate that some skinning procedures were carried out there. Similar explanations of the phalanges existence in archaeozoological materials were frequent in accessible literature [8,23,24,43,44,45]. The deficiency of trunk skeletal elements in the material from Kamienna Góra could probably be explained by horse butchery. There are no larger elaborations of any consumptive animal wastes from this area. Simultaneously, the archaeozoological investigations of animal bone assemblages form Wroclaw or Opole (two of the most municipal centers of the Silesian province), the lack of frequent horse butchery evidence, it therefore seems rare and uncommon to be used in Silesian cuisine [43,44,45]. The horse slaughter and horses’ meat consumption were prohibited by the Roman Catholic Church in the Middle Ages. Deschler-Erb, and Stopp [24] stressed that early modernity brought some changes, and the spirit of relationalism in this historical period, allowed for the horse to be used as a slaughter animal. The differences visible in the distribution of anatomical elements of horses’ bones form Kamienna Góra, Jelenia Góra, and Złotoryja can be explained by other attitudes towards horsemeat in these three Silesian towns. Moreover, the lack of any horse’s skull findings can prove that horses were not slaughtered by executioners in all three towns. On the other hand, Deschler-Erb and Stopp [24] suggested that the size and body mass of horses and their cadavers meant that these animals could be easier brought to the knacker’s yard and killed there, rather than transporting the heavy cadaver from another place. He also stressed, that written sources of A. Parent-Duchâtelet from 1836, gave us the descriptions of a possible slaughter method (blunt hit to the head or heart pierce). The clear proof of the executioner’s responsibility for animal flesh utilization, with the municipal council’s decision sent out in Szprotawa (Ger. Sprottau) in 1736, obliged the executioner to dead cattle cadaver (plague) gatherings and depositions in the deep pits, which were additionally secured from wild animal access [28]. It is interesting that the municipal authorities allowed knackers to take edible meat. The latter assumption must be controversial for modern veterinarians. The contained meat acquired from dead animals is a proven source of danger for both animals and humans. The only explanation of this procedure is the lack of necessary knowledge on epidemiology and zoonoses. Partly, this phenomenon could be the result of Catholics and Protestants uneasy coexistence, still vivid in the 17th and 18th centuries or caused by the geographical location and economic conditions (Kamienna Góra-Sudetes Mountains; Złototyja-Katzbach Foothills). Similar geographical differences between western and eastern Switzerland were observed in the mentioned historical period [24]. The use of horse’s flesh as a food for hunting dogs due to the urban character of investigated sites can be excluded [56].

The strong fragmentation of bone material made any assumptions on animal killing methods impossible. Likewise [24], the horses were possibly killed within the knacker’s yard, because of the size and weight of the animals. The existence of horses’ phalanxes allows for the statement that the animals were skinned by the knackers, too. Horse carcass skinning was (likely) especially intensive in Jelenia Góra, where the excess of horse phalanxes were clearly visible in the distribution of anatomical elements. Rare signs of cut and chop marks together with gnawing marks are presented in Table 8.

Although no medieval or early modern tannery discovered by archaeologists was mentioned in this article, it is possible to prove a rare use of horse’s leather in medieval Silesia [57]. The literature provides the information that the dominating sources of animal skin were domestic animals (cattle, small ruminants, and pig) [24]. Deschler-Erb and Stopp [24] stressed that a horse’s skin tannery proof was rare in both Medieval and Early Modern archaeology, due to the possible but heavy procedure of horse skin elaboration in contrast to other species.

The horse’s bone material form Letzigraben (Zürich-Albisrieden), reported by Deschler-Erb and Stopp [24], was found in anatomical order and without larger cut or chop marks, which can be observed due to skin, hoof, or meat acquisition by humans. The horse’s skeletal remains form all three investigated sites were strongly fragmented and poorly preserved. Animal carcasses were utilized within knackers’ yards and stored directly in the neighborhood of gallows. The animal wastes were deposited in animal catcher waste pits (i.e., fully unearthed pit no. 1/13) or inside the construction of gallows. Periodically, the knackers’ yards and the areas around nearby gallows were cleaned up [28]. The above-mentioned factors strongly influenced the animal remains preservation status.

The distribution of anatomical elements (body parts) analyses of canine skeletal remains confirmed the predominance of appendices, which can be explained by strong fragmentation of the investigated material in comparison to other studies on less fragmented consumptive bone wastes [43,44,45]. The deficiency of phalangeal elements could be due to their size (omission), and this factor was important for animal waste translocation among dogcatcher activity areas in the past. The predominance of equine and canine bone remains proved that the animals mentioned were the main interest of the animal catcher at this site, which resembles results of other studies [8,23,24]. The lack of cynophagia can be excluded, not only because of the lack of any bone marks, typical of consumptive wastes, but also from the cultural and religious point of view [24].

The distribution of anatomical elements, forming the body parts, methodology introduced by Lasota-Moskalewska [37] brought the possibility of the under- or over-representation in the subsequent number of body parts. The existence of dental remains within the alveoli and separated teeth can be a good example of the disadvantages of this method, and should be considered when referring to this research. The strong fragmentation of cranial skeleton, together with teeth removed from the alveoli, can significantly change the proportions between the number of bones belonging to the head and postcranial skeleton. Moreover, the long bones can be easier fragmented than the short bones (carpal, tarsal bones or phalanges) and the latter are not only numerous, but also small (not complete collection achieved during site exploration or secondary deposition in the past during translocation). These inconveniences shall be taken under consideration in the archaeozoological analysis and conclusion. Moreover, the first and second order changes significantly influence the quality and quantity of unearthed skeletal remains assemblages [8]. During our investigation, the animal catcher’s waste pit was discovered and fully explored. All accessible bone remains were analyzed and achieved results seem to be comparable to these, which were obtained using the archaeozoological material of all three gallows. We have to remember that strong fragmentation of artifacts, potential gatherings, and re-depositions in the past can significantly influence the conclusions.

The minimum number of individuals is a valuable tool for comparison of the frequency in different species. Unfortunately, the preservation status and fragmentation of bone assemblages, together with its complex history (i.e., taphonomic factors) made the estimation of MNI difficult, or even impossible, and further conclusions unreliable. The investigated skeletal material could come both from complete and incomplete carcasses. Some information of animal use and utilization by knackers and dogcatchers provide useful information relating to the species frequency and distribution of anatomical elements.

Moreover, Stampfli [23] noted other researchers’ findings relating to the animal skeletal remains weight estimation, and its usefulness for further conclusions to be made about animal herd quantity and quality, as being strongly related to animal body constitution, carcass preservation, skeletal remains, humidity etc. We fully agree with this assumption, stressing that animal health status, body build (animal constitution), and environmental factors (animal condition) are able to radically influence the animal bones remains from the past.

Finally, the small percentage of bones from cattle, sheep, goats, and pigs did not allow any conclusions to be made concerning these domestic animals. The investigated assemblages do not deliver relevant data for drawing conclusions, as opposed to assemblages from other Silesian cities representing typical consumptive wastes [43,44,45]. Horses could be used as draught animals, but this method of animal use caused no bone changes in the majority of cases, so the animals must have been used in a rational and careful way. In some cases, we found advanced dental deformations.

The investigated bone assemblages did not contain any skeletal remains from animals that were not anatomically adult. The dentition analysis proved that the horses in all three sites, Kamienna Góra, Złotoryja, Jalenia Góra, could be described as adult and mature animals. Similar results were achieved by Emmenbrücke Stampfli [23]; the only difference was the lack of animals below 2 and above 20 years old. The age estimations of horses from Zürich-Albisrieden showed that the animals were 15–18 and above 20 years old [24]. This seems to exclude mass slaughter and animal death caused by breeding errors, lack of fodder, chronic diseases, or lack of care in these human communities. The horses aging excluded the use of very old animals. The accessible material did not allow for any sex differentiation. The domestic dog remains came from anatomically adult animals. The routine duty of executioners included the capture and elimination of stray animals [23,28,42,49]. The puppies were less important, and even if they were of interest to a dogcatcher, their skeletons would not have been fully ossified (far less likely to be preserved than the skeleton).

Animal pathologies are not always visible as bone changes in the skeletal system. Due to morphological conditions of investigated animal skeletal remains, we can speculate that the majority of the animals were healthy. Any visible signs on the bone structures could have been caused by advanced age or rarely intense draught animal use. Acute diseases cannot be excluded, despite a lack of evidence of bone changes that would be typical of such conditions. The changes of bone morphology were marginal in investigated animal skeletal remains assemblage (below 1.7% of NISP), presented in Table 9.

Marginal intake of bone changes indicates animal overloading, chronic disease symptoms, or lack of malformations visible in archaeozoological analysis, allowing for hypothesizing that the health status of the majority of the animal population was good. Rare bone changes were mainly age related. It could be explained that the animals were less intensively used, not inbred, and free from chronic contagious and contentious diseases, but it should be also stressed that the preservation of the skeletal remains (the more intensive influence of the taphonomic factors) and NISP was lower than in other scientific reports [23,58]. The results of palaeopathological investigation in Zürich-Albisrieden were interpreted as proof of advanced age, being kept in bad conditions and the lack of proper animal use [24].

The pathological changes identified in bone assemblages from Kamienna Góra and Złotoryja will be further analyzed and presented in separate work.

### 4.2. Written Sources

**Figure 6:**
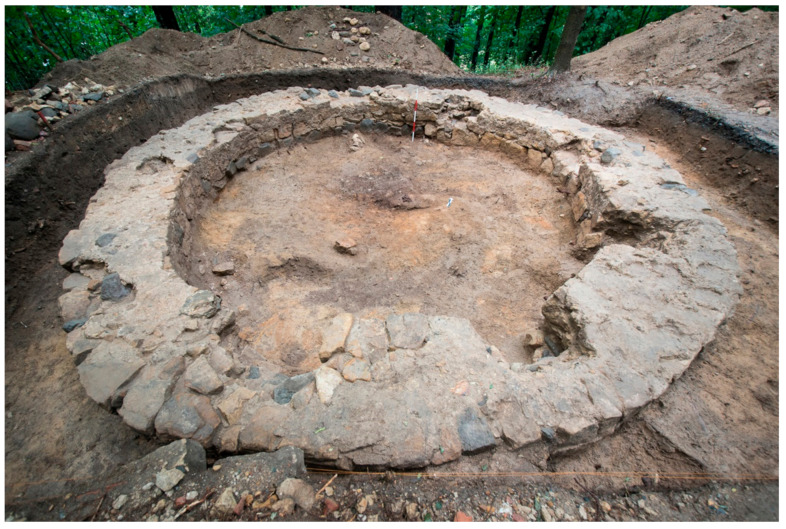
Unearthed gallows buttress at Złotoryja site. Photo by M. Mackiewicz.

Traditional gallows were typical features of the Silesian suburban landscape until the 19th century. Sometimes larger villages had such places, but to gain some local prestige rather than to use them as intended [39]. The majority of execution places were surrounded not only by dishonored human cemeteries, but also dog catcher waste pits, also known as fields (German: Schindergrube, Schinderanger, Schinderplatz), as executioners were also responsible for the disposition of bodies or remains of stray, useless, or dead animals. Both human and animal skeletal remains were treated similarly, without any esteem [24,25,26,27,28,41]. Archaeological explorations of execution places in early modern Silesian towns revealed that the central point of such facilities was a cylindrical well-like brick construction with three or four pillars and a crossbeam at its crown (Figure 6).

The scaffold could be up to 6.5–7 m high, and it was usually located outside the town walls [28]. The location and function of the gallows were usually restricted by law, which reflected not only their warning function, but also practical thinking about early hygiene.

Sometimes the juridical rules were not strictly obeyed by the executioners. A good example of such practice was described on 16 February, 1733 in Wrocław, when a suicide victim or a person who had committed suicide was not buried in the municipal gallows neighborhood, but in another place, near the city gate that was an animal waste pit. This was probably easier and faster for the executioner [28].

Such negligence of the executioner’s duties was also noted for animal utilization. Sometimes the waste pits were not deep enough or were neglected. Then dogs or other animals could dig up animal remains and spread them inside or outside the town. These problems were usually solved by municipal decisions, sometimes also by a pay rise to encourage the executioners to work harder [24,28,40,41].

Animal catching and utilization were strictly connected to a municipal executioner’s duties [49]. Usually, these professionals were assisted by peon responsible for less important tasks [24,26,28,41]. In Silesian towns, there were sometimes many gallows. Juridical orders sent by municipal councils were often overlooked, and the prolonged destruction of animal and human cadavers left without proper care in waste pits was a frequent reason for the intervention of the town authorities [28].

Human life in the neighborhood of gallows was burdensome, due not only to routinely carried out human executions and burials, but also to the existence of animal cadavers and animal catcher waste pits, which occupied large areas outside the town walls. At the beginning of the 17th century, a local executioner employed in the Silesian town of Niemcza, had to pay two thalers for waste pit possession and use [28].

Historical sources proved that the discarding of animal flesh and skeletal remains usually consisted of their burning and scattering or being left in animal catcher fields or intentionally dug pits [28,41]. The executioners were often able to gain additional profits, by developing trades linked to animal slaughter, to their basic duties. The majority of executioners engaged in animal fat and leather trading; therefore, the animal catcher workshop must have been sufficiently equipped and organized. Due to hygienic and practical concerns, it was considered ideal if the gallows could be located outside the city walls and in close proximity to a source of water. The executioner disposed of animal flesh by fire and burial; therefore, such places were known in German as *Richt und Cadaver Platz* [28,41]. Moreover, the executioner was responsible for some traditionally unpleasant works such as cleaning sewage systems, prisons, wells, and towers [28].

The modern progress of Enlightenment ideas and the introduction of human rights in the legal system significantly changed both juridical punishment methods and animal waste utilization solutions. Franz Friedrich Nessel, an executioner in Wrocław, the Silesian capital city, suggested in 1770 and 1774 that the current location for animal waste utilization was too small, and asked for a new and larger one. The gallows in various towns in the 18th century were transformed into large dog collection areas [28]. The time of executioners and gallows faded away. Human population growth and increased animal migration connected with trade intensity transformed the needs of the modern world in relation to the animal welfare and veterinary care.

The next chapter of animal care, control, and utilization belonged to veterinarians, and it was accompanied by modern ideas of food hygiene from pitchfork to fork and the establishment of veterinary schools. The beginning of modern veterinary education is set at year 1762, when Claude Bourgelat established the first European school of veterinary medicine in Lyon. Even that veterinary education primary was devoted only to military needs (cavalry horses), the development of veterinary schools in the countries of 18th and 19th century Europe produced fully qualified veterinarians. Earlier attempts of professional supervision on animal health statuses, animal care, and food hygiene carried out by human medicine doctors appeared to be ineffective. The departments of veterinary, founded within the existing faculties of medicine, were unable to give sufficient knowledge and the necessary skills to human physicians (who worked as municipal physicians), and their functions were transformed into the source of information about anthropozoonoses in medical curriculum [17,18,29]. The division of biological sciences into subsequent branches, such as human and veterinary medicine, biology, ecology, etc., was developed in the 18th century. From ancient times, scientists explored the animated nature, becoming, simultaneously, interdisciplinary experts. Modernity gave rise to the schools of anatomy (*theatrum anatomicum*), which played an important role in the development of the morphological sciences, before departments of anatomy formed within universities [17,18]. The archaeological evidences of such activities in the Royal London Hospital proved that the human–animal relations were different from today and dissected animals were frequently buried together with human body parts [59]. We have to remember, that the human–animal–environment relationship underwent dynamic changes in the past, especially in post-medieval and modern Europe [60,61]. The animal role, care, and usage used to determine its social status. The upper classes treated their favorite horses, dogs, or cats better than servants; the pet animals did not need any practical function and became simply a member of the family [60]. Such social status of a pet animal strongly contrasted with Cartesian’s theory of ‘beast-machines’, which excused the brutality towards experimental animals in early modern sciences [61]. The human curiosity and development of modern sciences allowed for vivisections. Well-educated rich noblemen or enlightened scientists organized curiosity cabinets and studied animal morphology, whilst at the same time, the same people usually had their beloved animals [60,62]. This phenomenal dichotomy in ethics and rationalism is still a problem now. The interpretation of archaeozoological materials based on rational criteria allows for differentiation between the skeletal remains of domesticated livestock, stray animals, and pet animals [62,63]. The context of deposit, butchery marks, pathologies, or animal age indicated that investigated bone remains form Silesian gallows and knackers’ yards show typical signs of animal remains utilization and potential fur or fat use with different intensity in all three towns. To summarize, the human–animal relationship was far from the humane treatment typical for pet animals and clearly reflects the knacker, animal catcher, and executioner practices. Archaeozoological signs of human intentional activities, animal pathologies fragmentary visible in bone material, together with socioeconomic factors, religion, individual treatment of animals, archaeological context, etc., would allow a definition of animal ‘care’ and animal ‘abuse’, if only the links between them were found [64].

Due to the wideness of this topic, we had to narrow our interest to Silesia. The first institutional regulations of animal trade, health control, and food hygiene supervision were sent out during Germany rule (Kingdom of Prussia) in 1844. The same country introduced the obligatory trichinoscopy in 1875. The rise of the German Empire spread the uniform food hygiene law in 1878. Finally, the discoveries of Robert von Ostertag and many others, in his work entitled: ‘*Lehrbuch für Fleischbeschauer’*, became the source of rules, supervision system construction, and veterinarian responsibilities later introduced in the whole of modern Europe [29].

The animal care and all aspects of zoonoses prevention and prophylactics became the part of veterinary medicine education in the modernity as a direct result of the scientific interests of Robert Koch, Erik Viborg, Bernhard Laurits Frederic Bang, Maurice Nicolle, Adilo Mustafa, and many others. One of the crucial points of veterinary medicine development was the invention of the rabies vaccine by Louis Pasteur and Pierre Emile Roux in 1885, as the first step to successfully defeat this horrific disease, for which the mere mention of the name and suspicion caused fear even between executioners [18].

## 5. Conclusions

Our study shows significant differences in species and distribution of anatomical elements of bone assemblages from investigated knackers’ yards and classical post-consumptive animal skeletal remains from inhabited areas. It should be stressed that the archaeozoological analysis proved that the skeletal remains probably came from healthy adult animals, but we have to remember that not all animal diseases are reflected on skeletal system morphology. Therefore, some of them remain unrecognized.

Moreover, the accessible literature and historical sources, together with the archaeozoological findings, proved the important role of executioners in early modernity. Animal population control, animal collection areas, and animal waste utilization were important parts of this profession and sources of additional financial income.

The beginnings of modern veterinary education together with the introduction of human rights into the law were crucial for the formation of a recognizable animal trade, healthcare, control, and utilization system. The municipal executioners vanished with the gallows.

Apart from the complicated nature of the executioner’s duties; his role in early municipal animal control, and the development of veterinary supervision on infectious diseases, food sanitary law, and animal welfare, the archaeozoological analysis provides some proof of the role of the human–animal relationships in the past. Although this study cannot fully provide the answers, due to the poor preservation status of accessible material, some useful information can be collected and used to reconstruct the human life landscape between the gallows fading and the rise of animal care in the modern meaning of this word.

## Figures and Tables

**Table 1:** Percentage of bone remains with absolute values at investigated gallows. N—number of specimens, TNF—total number of fragments, number of identified specimens (NISP), burned—burned specimens (unidentified and identified), unidentified—number of unidentified specimens. Kamienna Góra, site—all accessible bone material assemblage form this site, object no. 1/13—skeletal remains from knacker’s pit located within the site.

Archaeological Site	Złotoryja	Kamienna Góra		Jelenia Góra
		Site	Object no. 1/13	
	N	N	N	N
TNF	9013	9037	5459	1207
NISP	3626 (40.23%)	2253 (24.9%)	1083 (19.83%)	639 (52.95%)
Burned	82 (0.9%)	2405 (26.7%)	2019 (36.98%)	332 (27.5%)
Unidentified	5387 (59.77%)	4379 (75.1%)	4376 (80.17%)	568 (47.05%)

**Table 2:** Frequency of taxa in NISP, archaeological site at Złotoryja, Kamienna Góra and Jelenia Góra. *Bos*, cattle; *Sus*, swine; *Ovis*, sheep; *Ovis*/*Capra*, sheep/goat group; *Equus*, horse; *Canis*, dog; *Canis*/*Felis*, dog/cat group; *Canis*/*Canis lupus* dog/wolf group; *Felis*, cat; *Canis lupus*, wolf; *Vulpes*, fox; *Sus scrofa*, wild boar; *Sus*/*Sus scrofa*, swine/wild boar group; *Talpa*, mole; *Mus*, mouse; *Rattus sp.*, rat; *Lepus*, hare; *Rodentia*, rodents; *Homo*, human; *Gallus*, hen; *Anser*, goose; *Aves*, birds; *Corvus*, crow; *Gastropoda*, snail. Kamienna Góra no. 1/13 present the NISP estimated in skeletal material from the complete animal-catcher waste pit unearthed within the Kamienna Góra gallows site.

Taxon	Złotoryja	NISP%	Kamienna Góra	NISP%	Jelenia Góra	NISP%	Kamienna Góra No. 1/13	NISP%
*Bos*	175	4.83	83	3.68	24	3.82	52	4.80
*Sus*	140	3.86	55	2.44	16	2.55	25	2.31
*Ovis*	1	0.03	1	0.04	0	0.00	1	0.09
*Ovis/Capra*	272	7.50	26	1.15	14	2.23	11	1.02
*Equus*	1568	43.24	1232	54.68	413	65.76	619	57.16
*Canis*	998	27.52	796	35.33	145	23.09	357	32.96
*Canis/Felis*	2	0.06	0	0.00	0	0.00	0	0.00
*Canis/Canis lupus*	1	0.03	0	0.00	0	0.00	0	0.00
*Felis*	11	0.30	5	0.22	2	0.32	1	0.09
*Canis lupus*	0	0.00	2	0.09	0	0.00	0	0.00
*Vulpes vulpes*	170	4.69	15	0.67	2	0.32	10	0.92
*Sus scrofa*	0	0.00	2	0.09	0	0.00	0	0.00
*Su/Sus scrofa*	4	0.11	1	0.04	0	0.00	0	0.00
*Talpa europaea*	1	0.03	0	0.00	0	0.00	0	0.00
*Mus musculus*	4	0.11	0	0.00	0	0.00	0	0.00
*Rattus sp.*	4	0.11	0	0.00	0	0.00	0	0.00
*Lepus lepus*	2	0.06	0	0.00	1	0.16	0	0.00
*Rodentia*	5	0.14	2	0.09	0	0.00	0	0.00
*Homo*	216	5.96	27	1.20	4	0.64	6	0.55
*Gallus*	15	0.41	0	0.00	1	0.16	0	0.00
*Anser*	6	0.17	1	0.04	5	0.80	0	0.00
*Aves*	29	0.80	5	0.22	1	0.16	1	0.09
*Corvus corax*	1	0.03	0	0.00	0	0.00	0	0.00
*Gastropoda*	1	0.03	0	0.00	0	0.00	0	0.00
NISP	3626	100.00	2253	100.0	628	100.00	1083	100.00

**Table 3:** The minimum number of individuals (MNI) estimated for horses and dogs from early modern Silesian gallows.

Site	Kamienna Góra		Złotoryja		Jelenia Góra	
taxa	horse	dog	horse	dog	horse	dog
MNI	6	66	13	65	10	14

**Table 4:** The osteometry of measurable dog and horse bone remains from Kamienna Góra with computed height at withers [mm]. The abbreviations after A. von den Driesch (1976). G—greatest length of bone, GLI—greatest lateral length of bone, GLC—greatest length form head of bone, GLP—greatest length of articular process, TPA—depth of anconeal process, KLC—smallest length of scapular neck, KTO—smallest depth of olecranon, BPC—width of coronoid processes, LO—length of olecranon, LI—lateral length of bone, Bp—greatest width of proximal extremity, Tp—greatest depth of proximal extremity, LG—length of glenoid cavity, BG—width of glenoid cavity, Td—greatest depth of distal extremity, KD—smallest width of diaphysis, Bd—greatest width of distal extremity, W_height_—height at withers, W_height_ mean—height at withers mean value.

	Dog									Horse	
Measurement	Humerus	Radius			ulna	Femur	Tibia			Scapula	Metacarpus
GL	99.11	144.48	87.91	87.91	103.63		100.07	98.22	157.95		250.04
GLI											249.86
GLC						104.39					
GLP										101.45	
TPA					19.29						
KLC										72.49	
KTO					16.36						
BPC					11.76						
LO					20.98						
LI											245.91
Bp	15.01	16.71	13.23	13.24		17.16	24.47	26.71	24.81		54.07
Tp											37.45
LG										63.58	
BG										50.49	
Td	22.19										
KD	7.21	10.97	9.68	10.16		7.42	9.85	10.38	10.48		35.96
Bd	19.43	21.67	18.15	17.44		18.61	15.95	17.57	17.57		41.56
W_height_	323.9	465.2	283.1	277.2	276.7		292.2	286.8	461.2		1331.8
W_height_ mean	333.3									1331.8	

**Table 5:** The osteometry of measurable horse’s bone remains from Złotoryja with computed height at withers [mm]. The abbreviations after A. von den Driesch (1976). GL—greatest length of bone, GLI—greatest lateral length of bone, Bp—greatest width of proximal extremity, greatest depth of proximal extremity, BFp—greatest width of proximal articular surface, Td—greatest depth of distal extremity, TD—smallest depth of diaphysis, KD—smallest width of diaphysis, Bd—greatest width of distal extremity, BFd—greatest width of distal articular surface, W_height_—height at withers, W_height_ mean—height at withers mean value.

	Horse							
Measurement	Radius	Metacarpus			Tibia	Metatarsus		
GL	319.99	224.69	215.65	218.08	347.81	280.05	239.44	232.81
GLI		219.93	211.92	214.08		275.89	230.52	229.47
Bp	311.32	48.33	46.19	51.26	89.99	52.97	37.77	45.65
Tp		32.31	28.39	33.74		42.59	32.93	36.48
BFp	70.81							
Td		32.04	32.52	46.63	41.31	37.64	32.57	32.94
TD		24.15	19.48	22.42		30.89	22.23	23.11
KD	64.85	33.41	25.98	31.38	38.88	34.99	26.92	28.65
Bd	67.19	46.06	46.22	33.88	68.34	52.82	40.72	44.19
BFd	56.59							
W_height_	1279.9	1409.8	1358.4	1372.3	1372.8	1492.7	1276.2	1240.9
W_height_ mean	1350.4							

**Table 6:** The osteometry of measurable dog’s bone remains from Złotoryja with computed height at withers (mm). The abbreviations after A. von den Driesch (1976). GL—greatest length of bone, GLC—greatest length form head of bone, Bp—greatest width of proximal extremity, Tp—greatest depth of proximal diaphysis, TC—smallest width of diaphysis, Bd—greatest width of distal extremity, W_height_—height at withers, W_height_ mean—height at withers mean value.

	Dog							
Measurement	Humerus						Femur	
GL	190.87	165.19	134.41	127.17	89.63	117.44		
GLC	186.23	160.37	130.08	125.47	87.79	115.59	148.82	156.91
Bp	34.17	29.37	33.85	22.35	21.04	19.19	32.51	32.68
Tp	48.78	39.85	27.33	31.29	25.91	27.91		
TC							14.92	16.17
KD	18.58	12.63	12.99	10.49	9.26	9.19	10.97	11.09
Bd	41.88	33.58	27.86	25.91	22.87	22.87	26.04	27.71
W_height_	643.2	556.7	452.9	428.6	302.1	395.8	447.9	471.7
W_height_ mean	461.5							

**Table 7:** Distribution of anatomical parts of horse and dog skeletal remains. Normal, normal anatomical percentage of skeletal fragments.

	Dog				Horse			
	Normal	Kamienna Góra	Złotoryja	Jelenia Góra	Normal	Kamienna Góra	Złotoryja	Jelenia Góra
head	20	14.9	13.4	14.4	23	26.4	13.1	6.7
trunk	40	41.1	35.2	54.8	43	25.8	61.6	48.8
appendices proximal	7	12.3	18.7	11.0	7	3.3	3.5	6.7
appendices distal	15	21.8	22.8	14.4	21	31.1	17.0	17.4
phalanges	18	9.8	9.8	5.5	6	13.4	4.7	20.5

**Table 8:** The summary of bone marks identified in animal skeletal remains from Kamienna Góra and Złotoryja gallows.

No.	Signature	Taxa	Bone	Marks
1	KG 1/13	Equus	phalanx I	cutting
2	KG trench II	Equus	femur	chopping
3	M/45/M	Equus	thoracic vertebra	chopping
4	M/87/Z	Canis	femur	chopped off head of femur
5	M/87/Z	Sus/Sus scrofa	humerus	gnawing
6	M/16/Z	unidentified	ulna	chopping
7	KG trench II	Equus	thoracic vertebra	chopping
8	KG trench II	Bos	metapodium	chopped off
9	KG trench I	Bos	tibia	chopped off
10	KG trench II	Bos	costa	cutting
11	KG trench II	Equus	humerus	chopping
12	KG trench I	Bos	femur	chopped off lesser trochanter

**Table 9:** Summary of bone pathologies caused by many different factors.

No.	Archaeological Site	Species	Bone Fragment	Pathology Type	Cause
KG W2	Kamienna Góra	dog	*radius sinister*	new bone formation surrounding the osteolysis area	unknown
KG W2	Kamienna Góra	dog	*ulna sinistra*	new bone formations nearby the margin of articular cartilage of anconeus process, trochlear notch and lateral coronoid process	age related bone changes
KG W2	Kamienna Góra	dog	*femur sinister*	new bone formation at the neck of femur nearby the articular surface and within the trochanteric fossa	age related bone changes
KG W2	Kamienna Góra	horse	*os ungulare*	new bone formations at the palmar processes	age related bone changes, ossification of the ungular cartilage
KG W1/2	Kamienna Góra	horse	*sesamum ungulare*	new bone formation in the flexor surface of navicular bone and in the middle of its free margin	probably animal overloading
KG W2	Kamienna Góra	dog	*vertebra lumbalis*	new bone formations in the lateral and ventral surfaces of vertebra body	age related bone changes, spondylosis
KG W2	Kamienna Góra	horse	*dentes buccales*	deformation of the occlusal surface of tooth	malocclusion
4/16	Złotoryja	horse	*vertebrae lumbales*	two fused lumbar vertebrae	animal overloading, spondylosis
M/16/Z	Złotoryja	horse	*ossa carpalia*	new bone formations	bone spavin
M/51/Z	Złotoryja	horse	*ossa carpalia*	new bone formations	bone spavin
M/138/Z	Złotoryja	horse	*ossa tarsalia*	new bone formations	bone spavin
M/76/Z	Złotoryja	horse	*vertebrae thoracicae*	new bone formation at the attachment points of ventral longitudinal ligament	young animal overloading and intensive use
M/45/Z	Złotoryja	horse	*vertebrae thoracicae*	new bone formations caused by chronic inflammation	unknown
M/40/Z	Złotoryja	dog	*vertebrae lumbales*	new bone formations nearby the cranial articular processes	age related bone changes

## Data Availability

Not applicable.
